# Mapping the FtsQBL divisome components in bacterial NTD pathogens as potential drug targets

**DOI:** 10.3389/fgene.2022.1010870

**Published:** 2023-01-04

**Authors:** Harbinder Kaur, Andrew M. Lynn

**Affiliations:** School of Computational and Integrative Sciences, Jawaharlal Nehru University, New Delhi, India

**Keywords:** neglected tropical disease, divisome, FtsQBL, remote homologs, profile HMMs, AlphaFold

## Abstract

Cytokinesis is an essential process in bacterial cell division, and it involves more than 25 essential/non-essential cell division proteins that form a protein complex known as a divisome. Central to the divisome are the proteins FtsB and FtsL binding to FtsQ to form a complex FtsQBL, which helps link the early proteins with late proteins. The FtsQBL complex is highly conserved as a component across bacteria. Pathogens like *Vibrio cholerae*, *Mycobacterium ulcerans*, *Mycobacterium leprae*, and *Chlamydia trachomatis* are the causative agents of the bacterial Neglected Tropical Diseases Cholera, Buruli ulcer, Leprosy, and Trachoma, respectively, some of which seemingly lack known homologs for some of the FtsQBL complex proteins. In the absence of experimental characterization, either due to insufficient resources or the massive increase in novel sequences generated from genomics, functional annotation is traditionally inferred by sequence similarity to a known homolog. With the advent of accurate protein structure prediction methods, features both at the fold level and at the protein interaction level can be used to identify orthologs that cannot be unambiguously identified using sequence similarity methods. Using the FtsQBL complex proteins as a case study, we report potential remote homologs using Profile Hidden Markov models and structures predicted using AlphaFold. Predicted ortholog structures show conformational similarity with corresponding *E*. *coli* proteins irrespective of their level of sequence similarity. Alphafold multimer was used to characterize remote homologs as FtsB or FtsL, when they were not sufficiently distinguishable at both the sequence or structure level, as their interactions with FtsQ and FtsW play a crucial role in their function. The structures were then analyzed to identify functionally critical regions of the proteins consistent with their homologs and delineate regions potentially useful for inhibitor discovery.

## 1 Introduction

Cytokinesis is an essential step of cell division, and errors in this process may lead to cell death. To carry out this process accurately, bacteria employ a highly conserved and complex machinery known as the divisome. The divisome is a protein complex made up of more than 25 proteins (Du and Lutkenhaus, 2017), some of which are essential for cytokinesis. These essential proteins include early proteins (FtsA, FtsZ, ZipA), which form a proto-ring/Z-ring, and late proteins (FtsK, FtsQ, FtsL, FtsB, FtsW, FtsI, FtsN), which are recruited to the proto-ring (Aarsman et al., 2005; den Blaauwen et al., 2017; Söderström and Daley, 2017). Central to the divisome are the proteins FtsB and FtsL binding to FtsQ to form a complex FtsQBL, which helps link the early proteins with late proteins (Choi et al., 2018). Among the late proteins, FtsW and FtsI are critical components of peptidoglycan synthesis (Mercer and Weiss, 2002). FtsQ is essential and interacts with many other divisome components, which makes it an excellent target for cell division inhibitors (Buddelmeijer and Beckwith, 2004; Kureisaite-Ciziene et al., 2018). Both FtsQBL and FtsWI complexes are highly conserved across bacteria. Recruitment of the FtsWI complex depends on the cytoplasmic domain of FtsL (Gonzalez et al., 2010; Park et al., 2020) which is a component of the FtsQBL complex. Components of the divisome are excellent drug targets due to their essentiality. Highly homologous proteins would serve as targets for the design of broad host-range antibiotics, while remote homologs with more sequence divergence may serve as specific targets. The FtsW binds downstream to penicillin-binding proteins involved with peptidoglycan synthesis. These are well-studied and used antibiotic targets.

Neglected tropical diseases (NTDs) are prevalent in low-income economic regions of Asia, Africa, and the Americas. They are caused by diverse pathogens such as bacteria, viruses, protozoa, and helminths (Daumerie et al., 2010). Bacterial NTDs like Cholera, Buruli Ulcerans, Leprosy, and Trachoma are caused by pathogens *Vibrio cholerae, Mycobacterium ulcerans*, *Mycobacterium leprae,* and *Chlamydia trachomatis,* respectively. Cholera is a primeval disease that causes severe diarrhea due to the consumption of contaminated water and unhygienic living conditions (Faruque et al., 1998). The Buruli ulcer mainly affects the skin but can also affect bones, resulting in permanent disability (WHO, 2022a). The mode of transmission is not yet known, and treatment is costly, though it is believed to spread through personal contact. Leprosy is another chronic disease caused by the bacteria *Mycobacterium leprae* (WHO, 2022b). Many multidrug therapies are available, and the widespread global presence of the disease was reduced by 90% in three decades from 1985 (Kealey, 2010). But the treatment is not easily available to the very poor, and victims may continue to suffer from social stigma, disability, and disfiguration. Trachoma is an infection generally occurring in the eyelids caused by *Chlamydia trachomatis* (WHO, 2022c). It transmits through the discharge released from the eye of an infected person. Reinfection can occur and can result in visual impairment or complete blindness (Kealey, 2010). In comparison to other diseases, very few drugs have been discovered for NTDs recently (WHO, 2022c).

Interestingly, while *V. cholerae* has a self-sufficient genome contributing to its ability to survive in aquatic reservoirs outside of the host, *Mycobacterium* and *Chlamydia* have reduced genomes corresponding to their obligate intracellular parasitic nature (Stephens et al., 1998; Cole et al., 2001; Stinear et al., 2007). *Mycobacterium sp*. and *Chlamydia* seemingly lack many components of divisome which are shown to be essential in a model organism like *E. coli.* In *Mycobacterium sp*., some early proteins of divisome that helps in the correct assembly of FtsZ appear to be missing (Ouellette et al., 2020) until the sepF gene was identified (Gola et al., 2015), which interacts with mycobacterial FtsZ protein and alteration of which caused a division defect in M*ycobacterium smegmatis* that led to filamentous cells. *Chlamydia* has eliminated many unnecessary genes and processes and kept only the genes crucial for the bacterium to evolve into intracellular parasites. Surprisingly, some of the genes lost include a number of essential Fts cell division genes, including FtsZ (Stephens et al., 1998). In the past, (Ouellette et al., 2012) discovered and provided evidence that *Chlamydia* uses proteins that determine rod shape for cell division. They also proposed that MreB replace FtsZ in the division process. Following that, (Kemege et al., 2015) displayed MreB localization information at the cell division site for C*hlamydia*. Due to the deletion or replacement of these essential proteins, these pathogens have evolved a non-canonical divisome, with known homologs to canonical divisome proteins not detected using standard sequence similarity. The presence of FtsQ, FtsB, and FtsL homologs and their assembly into complexes in gram-positive bacteria like *Bacillus subtilis* and *Streptococcus pneumoniae* suggests that the FtsQBL subcomplex is evolutionarily conserved (Daniel et al., 2006). Beckwith’s group (2010) (Gonzalez et al., 2010) conducted a bioinformatics evolutionary analysis based on 16s rRNA sequences in 400 genomes and found that homologs of FtsQ, FtsB, and FtsL *E. coli* proteins were present in most of the organisms using a combination of HMMs with PFAM profiles and synteny. Results from this study also indicate the presence of all three proteins in the *Mycobacterium* and the presence of FtsL in *Chlamydia*.

These four bacterial NTDs represent genome diversity within bacterial phyla, including genome reduction. Ortholog mapping is an active area of research, with a growing number of methods that are chosen based on a user’s need for accuracy, speed, available computational resources, size of the application datasets or requirement for integration within a pipeline (Nichio et al., 2017). The Quest for Orthologs benchmark service provides a single framework to evaluate multiple publicly available methods (Nevers et al., 2022). Most methods are based on sequence similarity coupled with a higher-order graph or tree-based clustering for inference. There is a need for a standard method to propagate annotation from well-studied model organisms to identify orthologs irrespective of their sequence divergence. In this paper, a protocol is described to characterize distant remote homologs of *E. coli* proteins FtsQ (ecFtsQ), FtsB (ecFtsB), and FtsL (ecFtsL) in bacterial NTDs*.* The initial step for protein function prediction is often a sequence similarity search against the sequences of the known function. BLAST (Basic Local Alignment Search Tool) (Altschul et al., 1997) is widely used for sequence similarity search against non-redundant databases or customized databases. However, sequence-sequence comparison methods are unable to find the homologs in the target organisms that have very low sequence similarity. To search for the remote homologs of the FtsQBL complex of cell division proteins in *Mycobacterium* and *Chlamydia,* sequence-profile (Finn et al., 2011) and profile-profile Hidden Markov Models (HMMs) (Söding, 2005) was used.

The increased sensitivity of HMM-HMM methods comes at the cost of specificity-as remote homologs can share a common fold, but not necessarily common molecular function. These proteins can be differentiated either by sequence similarity to a homolog functionally characterized from an evolutionarily closer organism or from the protein structure. To more accurately predict the protein’s function and differentiate between proteins with a common fold, analysis of the protein’s structure can be added to the functional annotation pipeline. AlphaFold (Jumper et al., 2021) is an Artificial Intelligence (AI) based program for predicting protein structures with high accuracy and speed developed by Google’s DeepMind. Predicted structures of an ortholog will show conformational similarity with corresponding *E. coli* proteins despite having low sequence similarity. The fine function of proteins, which share almost the same domain organization leading to high structural similarity, can also be mapped from their structural characteristics. To distinguish between homologs that share a common structural motif or fold, their differential interactions with partner proteins in a complex could play an important role; FtsB and FtsL provide such a condition, being small bitopic amphipathic helices, each allowing sequence diversity without affecting their function, making them difficult to predict with accuracy using sequence similarity methods. The periplasmic region of both of them binds to the periplasmic region of FtsQ while the cytoplasmic region of FtsL binds to FtsW. To understand the interactions between the FtsQBL complex and the interactions between FtsL and FtsW, AlphaFold multimer (Evans et al., 2022) was used. Alphafold multimer predicts the structure for multi-chain protein complexes while maintaining intrachain accuracy of the structure. Structures of many components of the divisome from multiple bacteria are known, however, the structure of the entire complex remains unknown with few structures of interacting multimers. Crystal structures of FtsQ (2VH1) (van den Ent et al., 2008), the coiled-coil segment of FtsB (residues 28-63) (4IFF) (Lapointe et al., 2013), and FtsQB periplasmic complex (6H9N) (Kureisaite-Ciziene et al., 2018) from *E. coli* are present in the PDB database. As multimer models were used to predict function, the *E. coli* multimer structure was modeled with AlphaFold v2 and compared with known experimentally determined interaction to validate the method. Although FtsB and FtsL are unannotated in Uniprot for *M. ulcerans* and *M. leprae*, a previous study by Wu et al. (2018) have characterized FtsB and FtsL homologs for *Mycobacterium smegmatis*. These proteins were characterized with the help of HMM models and *in vivo* studies. Ouellette et al. (2015) characterized homologs of FtsQ and FtsL in *Chlamydia* with the help of bacterial Y2H assay, to date, these entries are still listed as hypothetical and uncharacterized in Uniprot along with FtsB homolog. The results from these studies were used to validate our approach.

## 2 Materials and methods

### 2.1 Data retrieval

The proteome sequences of all the four organisms, i.e., *Vibrio cholerae* (RefSeq ID: GCF_000016245.1), *Chlamydia trachomatis* (RefSeq ID: GCF_000008725.1), *Mycobacterium ulcerans* (RefSeq ID: GCF_000013925.1), and *Mycobacterium leprae* (RefSeq ID: GCF_000195855.1) were downloaded from the NCBI dataset. The complete genome filter in the NCBI dataset was applied and earliest completed genome was used for each organism in our analysis. Annotation and protein sequences of FtsQ, FtsB, FtsL, and FtsW homologs in *E. coli* were retrieved from the UniProt database (Bateman et al., 2021) with Uniprot ID P06136, P0A6S5, P0AEN4, and P0ABG4, respectively.

### 2.2 Identification of homolog and remote homologs

The remote homologs of the FtsQ, FtsB, FtsL, and FtsW from *E. coli* were identified in all the four organisms with the sequential use of BLAST (Altschul et al., 1997), HMMER (Finn et al., 2011), and HHSearch (Söding, 2005). The programs were installed locally using instructions from the distribution website. For BLAST, the *E. coli* sequences were used as query proteins, while the proteome files of the individual organisms were separately formatted as BLAST databases. For profile HMMs, ortholog profiles of query proteins from *E. coli* i. e COG1589 for FtsQ, COG2919 for FtsB and COG3116 for FtsL, were retrieved from EggNOG5 (Huerta-Cepas et al., 2019) and searched against target organisms’ protein sequences using “hmmsearch” from the HMMER version 3.2 package (Finn et al., 2011). Profile-profile mapping was carried out with the help of HH-suite3 package v3.0.3 (Steinegger et al., 2019). Multiple sequence alignment (MSA) profiles of query proteins and proteome files were generated with “HHblits” (Remmert et al., 2011) while performing two iterations with the Uniprot20 (version 2016), a clustered version of the UniProt database, which works well for ortholog detection. It may be noted that the default database currently distributed with HH-suite is the UniRef-30 and earlier Uniclust-30 (Mirdita et al., 2017), which performs well for the programs common use of detection of remote homologs at the fold level but not for our purpose of ortholog mapping. “HHmake” was used to convert the MSAs into profiles of the hhm format. A database was similarly built from the proteome file of target organisms *Chlamydia, Mycobacterium ulcerans, and Mycobacterium leprae.*

### 2.3 Structure prediction

AlphaFold v2.0 (Jumper et al., 2021) was implemented for structure prediction of the potential homologs for proteins FtsQ, FtsB, FtsL, and FtsW. AlphaFold was installed locally along with all the required genetic (sequence) databases using instructions from DeepMind’s GitHub repository (Jumper et al., 2021). A reduced version of all the databases (BFD, MGnify, PDB, Uniclust30, Uniprot, and UniRef 90) was used with the database preset option. The reduced_db preset has been optimized for speed and low hardware requirements. For individual proteins, AlphaFold monomer was used to predict the structure of remote homologs of FtsQ, FtsL, FtsB, and FtsW. To understand the multimeric interfaces to form the FtsQBL complex and recruitment of FtsW by FtsL, AlphaFold multimer (Evans et al., 2022) was used. AlphaFold Multimer requires a multifasta file as input. For generating both monomer and multimer models max_template_date = 2020-05-14 was used. Both models generated five models and ranked them on the basis of the plddt score.

### 2.4 Structure analysis

Structural alignment was performed between predicted structures of potential homologs with corresponding *E. coli* homologs using the STAMP alignment tool (Russell and Barton, 1992) from MultiSeq extension (Roberts et al., 2006) in VMD (Visual Molecular Dynamics) (Humphrey et al., 1996). Interactions between the multimeric protein complexes were calculated using the web server PDBSum (Laskowski et al., 2018). Images of superimposed structures of both monomers and multimers were generated using VMD (Humphrey et al., 1996).

### 2.5 Multiple sequence alignment and phylogenetic analysis

Orthologous sequences for each predicted homolog of FtsQ, FtsB, and FtsL proteins in all four organisms were extracted from the EggNOG v5 (Huerta-Cepas et al., 2019) database on the basis of the COG (clusters of orthologous groups) to which they belong. FtsQ and FtsB homologs mapped to COG1589 and COG2919, and two COGs were found for FtsL protein COG3116 and COG4839. The sequences corresponding to these COGs were downloaded, and the individual sequences were added for predicted homologs of A0PTJ5, O84041 and O84273 that did not map to any of the standard FtsB and FtsL COGs. A multiple alignment was built using the MAFFT v7.5 (Katoh and Standley, 2013) tool with default parameters. FastTree v2.1.11 (Price et al., 2010) was used to generate a phylogenetic tree from the multiple alignments using default parameters for protein sequences. A sequence logo was generated from MSAs with the help of Weblogo 3 (Crooks et al., 2004).

## 3 Results

### 3.1 Sequence similarity methods can predict potential candidate homologs in NTD bacterias with varying degrees of confidence

Homologs of ecFtsQ, ecFtsB, ecFtsL, and ecFtsW proteins were easily mapped in *V. cholerae* based on sequence similarity with BLAST. Single candidate proteins with low e-values (i.e., <0.001) (Table 1) were mapped as homologs to the *E. coli* query proteins. Probable peptidoglycan polymerase FtsW is highly conserved across bacteria and was also easily mapped using BLAST in all four organisms. For *Mycobacterium* and *Chlamydia*, a methodical study was used to find a remote homolog for FtsQ, FtsB, and FtsL as BLAST was not able to detect any significant hits for these proteins. The HMM-based method HMMER identified candidate homologs for FtsQ, FtsB and FtsL in *M. ulcerans* and *M. leprae* with significant e-value (i.e., <0.001) but was not able to provide any significant hit for *C. trachomatis*. Predicted FtsQ homologs in *M. ulcerans* A0PTI5 (muFtsQ) and *M. leprae* Q9CCE5 (mlFtsQ) (Table 1) are already annotated and present in Uniprot (Bateman et al., 2021) under the unreviewed annotation section. Both the homologs have slightly bigger sizes than the *E. coli* protein. The sequence-profile method identified two potential homologs for FtsB and one potential homolog for FtsL for each *Mycobacterium* sp. (Table 1). To identify the other proteins, a more sensitive method, profile-profile comparison, was used with a probability cutoff of 0.95 (HH-suite User Guide; Söding, 2005). This method successfully identified candidate proteins for remote homologs of ecFtsQ, ecFtsB, and ecFtsL in *Mycobacterium sp* and *Chlamydia*. The homolog identified as *Chlamydia* FtsQ (ctFtsQ) (Table 1) was annotated as a hypothetical protein in UniProtKB (O84769). Its sequence length (268 residues) is slightly shorter than the *E. coli homolog*.

**TABLE 1:** Results for FtsQ, FtsB, FtsL, and FtsW remote homologs mapped on target genomes. For BLAST and HMMER, only e-values are listed against the proteins. For HHsearch, both e-values and probability scores are listed.

Organism	Method	FtsQ	FtsL		FtsB		FtsW
*V. cholerae*		**Q9KPG9**	**Q9KPG0**		**Q9KUJ3**		**Q9KPG6**
	**Blast**	3e-56	4e-21		1e-21		2e-164
*M. ulcerans*		**A0PTI5**	A0PW54*	A0PTJ5*	A0PW54*	A0PTJ5*	**A0PTI8**
	**Blast**	-	-	-	-	**-**	1e-43
	**HMMER**	1.6e-40	2.5e-05	-	3e-26	1.5e-17	7.5e-50
	**HHsearch**	1.2E-29, 99.9	1.8E-15, 99.2	4.2E-11, 98.5	8E-18, 99.4	7.5E-13, 98.8	7.1E-58, 100
*M. leprae*		**Q9CCE5**	Q9CD41*	Q7AQC6*	Q9CD41*	Q7AQC6*	**Q7AQC4**
	**Blast**	**-**	**-**	**-**	**-**	**-**	3e-42
	**HMMER**	3.3e-40	3e-05	**-**	1.5e-23	4.7e-25	2.7e-49
	**HHsearch**	4.3E-30, 99.9	2E-15, 99.2	8.3E-12, 98.6	8E-18, 99.4	6.4E-13, 98.8	1.8E-63, 100
*C. trachomatis*		O84769*	O84041*	O84273*	O84041*	O84273*	**O84765**
	**Blast**	-	**-**	**-**	**-**	**-**	3e-36
	**HMMER**	-	**-**	**-**	**-**	**-**	5.6e-38
	**HHsearch**	2E-29, 99.9	7.4E-18, 99.4	2.1E-16, 99.3	9.3E-19, 99.5	4.4E-17, 99.4	1.1E-63, 100

The asterisk (*) sign indicates the proteins were unannotated in Uniprot.

The Bold text represents the Uniprot ids of candidate homologs and name of the Method used. This is purely stylistic and was used to distinguish them from numerical values.

Profile-profile methods identified two potential homologs each for both ecFtsB and ecFtsL in *Mycobacterium* and *Chlamydia*. These candidate homologs identified by both methods are same for FtsB and FtsL and hence these proteins are not significantly distinguishable using remote homology methods in both *Mycobacterium* sp. and *Chlamydia*. Although the size of these homologs is almost identical in *Chlamydia* compared to corresponding *E. coli* proteins, in *Mycobacterium sps.* it is much larger. It is reasonable to obtain ambiguous hits between these two proteins using profile-profile comparison algorithms, given that FtsB and FtsL are both small bitopic, amphipathic proteins with coiled-coil domains (Condon et al., 2018) and show low sequence conservation. Protein structure prediction was carried out to characterize these candidate remote homologs based on structural characteristics to solve the ambiguity between FtsB and FtsL.

### 3.2 Structural similarity is an additional validation of function inferred from remote sequence similarity

Candidate remote homologs of ecFtsQ, ecFtsB, and ecFtsL were identified using Sequence-Profile and Profile-Profile comparison methods. Although their sequences are not very close to those of *E. coli* proteins, when structural modeling was performed using AlphaFold, similar structures and conserved domains were detected.

**FIGURE 1:**
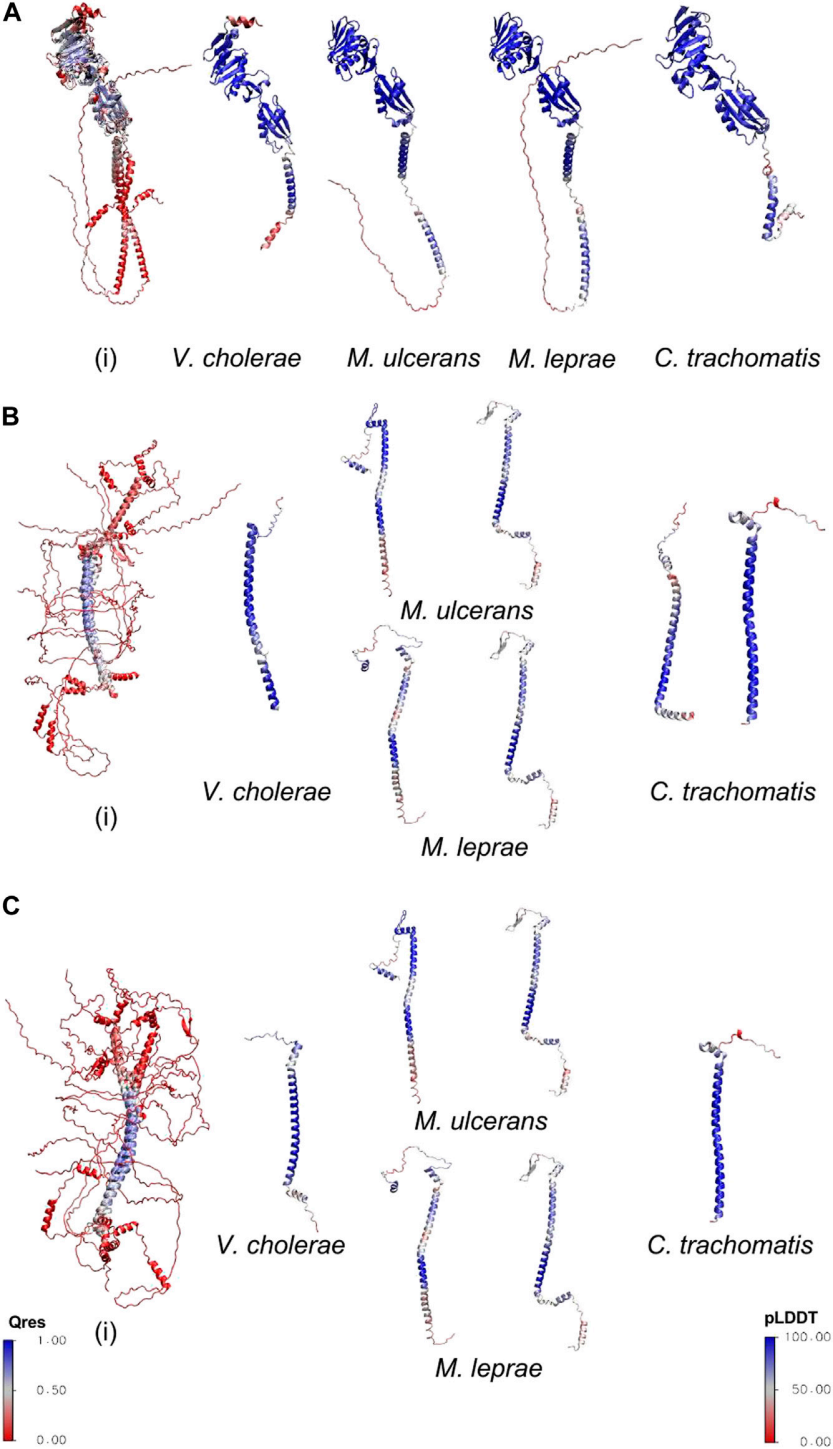
**(A)** The predicted structures of homologs superimposed with the experimental structure of *E. coli* FtsQ(PDB ID 2vh1). **(B)** The predicted structures of candidate homologs superimposed with FtsB homolog of *E. coli* (AlphaFold Model). **(C)** The predicted structures of remote homologs superimposed with FtsL homolog of *E. coli* (AlphaFold Model). The superimposed structures have been colored on the basis of structural conservation measures (Q-value) with blue being conserved and red variability between their structures. The individual structures of *V. cholerae, M. ulcerans, M. leprae*, and *C. trachomatis* are colored on the basis of AlphaFold confidence score.

FtsQ is a bitopic membrane protein composed of 276 amino acids and possesses the POTRA domain in the periplasmic region, which is crucial for its recruitment of binding partners (van den Ent et al., 2008). AlphaFold provides a confidence metric pLDDT which measures the accuracy of the predicted models. The models with pLDDT ≥ 90 represent prediction with high accuracy and between 70 and 90 represent a good backbone prediction (Jumper et al., 2021). The disordered regions were excluded from the calculation of the pLDDT score. The pLDDT score for *V. cholerae* FtsQ (vcFtsQ), muFtsQ, mlFtsQ and ctFtsQ are 89.9, 90, 84.7, and 88.30 respectively. The homologs for this protein in *Mycobacterium sps.* and *Chlamydia* are composed of almost the same number of amino acids: 317 and 268, respectively. Predicted FtsQ homolog for *M. ulcerans* shows a domain organization similar to *E. coli,* possessing a transmembrane domain (104-124 amino acids), and POTRA domain (128-196), but in contrast to *E. coli*, it also contains an extended cytoplasmic disordered region (1-61). Similarly, the ctFtsQ homolog contains a large periplasmic domain in addition to the periplasmic and transmembrane domain in the same conformation as of *E. coli* protein. In comparison to ecFtsQ, both homologs contain a similar conformation with the same number of helices and β-sheets in the periplasmic domain except for the last C-terminus helix. Structural alignment was carried out with STAMP (Russell and Barton, 1992) a tool integrated into the Mutiseq extension of VMD (Humphrey et al., 1996) to measure the overall structure conservation of these remote homologs. These models of homologs were superimposed with *E. coli* structure which was experimentally determined (PDB ID: 2vh1) (van den Ent et al., 2008) as shown in Figure 1A. The superimposed models of vcFtsQ and ctFtsQ have Qres values 0.7 and 0.5 respectively. The muFtsQ and mlFtsQ have 0.4 Qres value due to N-terminus disordered region. The periplasmic domain of predicted FtsQ homologs from *Chlamydia* and *Mycobacterium* exhibits significant structural conservation, which is crucial for forming a complex with FtsB and FtsL. However, there is some variation in the size of the last helix towards the C-terminus, which is followed by two β-sheets and residues that form the interacting surface with FtsB. This helix is truncated due to a deleted segment in *Mycobacterium* sp. and *Chlamydia* compared to the proteobacteria ecFtsQ and vcFtsQ.

FtsB and FtsL are both small bitopic proteins with a size of 121 and 103 amino acids in *E. coli*, respectively. Both proteins contain a coiled-coil structural motif–In all three organisms, HHsearch results show ambiguity in identifying FtsB and FtsL proteins. AlphaFold was used to predict structures of these detected homologs for FtsB and FtsL in all three organisms. The pLDDT measure for structural model of *V. cholerae* (vcFtsB) and *V. cholerae* (vcFtsL) are 85.5 and 84.3 respectively. In *Mycobacterium* sp. the structural models have large disordered regions and the confidence score was calculated excluding these regions. The confidence score for the candidate homologs for FtsB and FtsL is 81.8 and 81.4 in *M. ulcerans*; 81.03 and 72.8 for *M. leprae*. In *Chlamydia* the confidence score is 77.4 and 93.1 for the predicted homologs. Detected homologs of FtsB and FtsL in *M. leprae* and *M. ulcerans* are twice the sizes of ecFtsB and ecFtsL (Table 1) having large disordered regions in both N-termini (1-62 residues and 1-93 residues) as well as C-termini (205-227 residues and 350-377 residues) regions, respectively. Both the proteins possess a coiled-coil domain (118 and 145, 153-180 residues) and the transmembrane helical region (90-112 and 122-142). Similarly, *M. ulcerans* homologs also contain disordered N-termini (1-52 and 1-95 residues) and C-termini (199-233 and 213-328 residues). Coiled-coil (113-133 and 153-180 residues) and transmembrane regions (85-107 and 118-142 residues) are also present in both of the detected hits. In *Chlamydia*, the sequence length of predicted homologs for FtsB and FtsL is very close to *E. coli.* Still, due to similar domain organization, it is challenging to differentiate between FtsB and FtsL. Both the bitopic coiled-coil models contain the helical transmembrane (20-38 residues) region. To distinguish between FtsB and FtsL homologs for *Mycobacterium sps.* and *Chlamydia,* models of these candidate homologs were aligned with ecFtsB and ecFtsL (AlphaFold models) (Figures 1B, C) for all three organisms as the experimentally determined structure for the *E. coli* proteins has not been determined. The candidate homologs showed structural alignment with FtsB (Figure 1B). Similarly, FtsL showed structural conservation with all the candidate homologs in the *Mycobacterium*, but in *Chlamydia*, it showed structural alignment with only one protein (O84273) (Figure 1C). The overall Qres score for *Mycobacterium sps.* is low due to large disordered region on N and C termini but the coiled-coil domains show structural conservation. Based on these structural characteristics and alignment, it is difficult to distinguish between the remote homologs for FtsB and FtsL. FtsB and FtsL form a subcomplex and then bind to the periplasmic domain of FtsQ, and the cytoplasmic region of FtsL interacts with FtsW for the recruitment of FtsW to the septum site (Gonzalez et al., 2010). Interactions of FtsQB and FtsLW were studied from the modeled multimer complex of FtsK′, FtsQ, FtsL, FtsB, and FtsW to distinguish between the candidate homologs of FtsB and FtsL.

The structural model of FtsW, a polytopic membrane protein with ten transmembrane segments, is very similar across all the predicted homologs in all the organisms mentioned above. It shows minor variation in the first helical region (N-terminus) in *Vibrio* and *Mycobacterium*, and this helical region is absent in *Chlamydia*.

### 3.3 The *E. coli* FtsKQBLW complex serves as a reference for intermolecular interactions in bacteria

**FIGURE 2:**
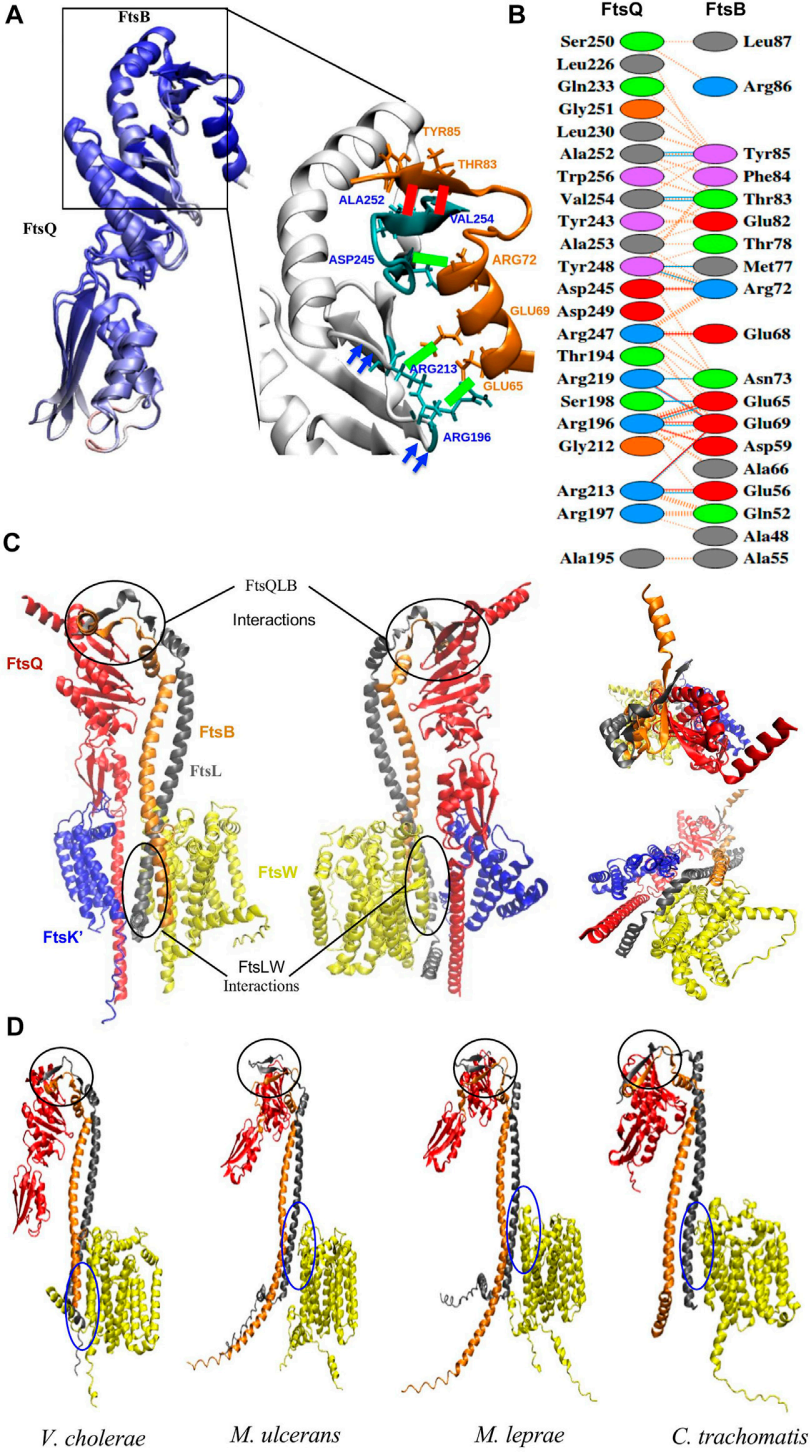
**(A)** Superimposition of the ecFtsQB PDB structure (6H9N) with the AlphaFold predictable model and are colored on the basis of Qres value. The black rectangle highlights the region of association between FtsQ and FtsB. The green colored bond represents the salt bridges and red colored bonds represents the Hydrogen bonds between two proteins. These interactions are conserved in both the structures. **(B)** PDBsum output shows the interfacing residues for modeled FtsQB from *E. coli*. The red, blue, and orange colored lines represent salt bridge, hydrogen bonds and non-bonded interactions. **(C)** Front, back, top and bottom view of the AlphaFold prediction model of *E. coli* FtsK’QLBW divisome subcomplex; individual proteins are represented in different colors: FtsK’ (blue), FtsQ (red), FtsL (gray), FtsB (orange), and FtsW (yellow). The black circles at the C-terminus highlights the binding regions between FtsQBL and towards N- terminus highlights those between FtsLW. **(D)** AlphaFold prediction model of the protein complex of FtsQ (Periplasmic domain), FtsL, FtsB, and FtsW in *V. cholerae, C. trachomatis, M. leprae, and M. ulcerans.* The black circle highlights FtsQBL and blue circle FtsLW binding regions respectively.

The predicted remote homologs for FtsB and FtsL are ambiguous in *Mycobacterium* sps. and *Chlamydia*. To differentiate these remote homologs as FtsB or FtsL, their selective interactions with FtsQ and FtsW respectively are pivotal (Gonzalez et al., 2010; Du and Lutkenhaus, 2017). The structure of the FtsQBL complex and the recruitment of FtsW by cytoplasmic FtsL is not fully understood. In *E. coli* FtsQBL protein complex occurs in stable conformations of trimeric (1:1:1) complex and hexameric (2:2:2) complex (Villanelo et al., 2011). So far, only the periplasmic FtsQ and FtsB subcomplex, have their bound structures determined experimentally (PDB ID 6H9N) (Kureisaite-Ciziene et al., 2018). AlphaFold multimer was used to predict the structure (Figure 2) of *E. coli* FtsK′, FtsQ, FtsL, FtsB, and FtsW to understand the interactions between these proteins. Three of these proteins (FtsQ, FtsB, and FtsL) each have a single transmembrane helix near their N-termini, while FtsW is a polytopic protein and contains 10 transmembrane segments and many loops within the cytoplasm that could interact with the cytoplasmic domain of FtsL (Pastoret et al., 2004). FtsQBL forms a complex independent of FtsK or FtsW. The transmembrane region of FtsK’ (up to 180 residues) was used to anchor the N-terminus of the FtsQ in the membrane, and to prevent this domain from interfering with the FtsBL interactions with FtsW.

Most of the critical interactions for the binding of FtsB and FtsL occur in the periplasmic region of FtsQ. In the model, the C-termini of both FtsB and FtsL form a strand-like structure only when bound to FtsQ. The AlphaFold modeled FtsQB was superimposed on to the crystal structure and was colored on the basis of Qres value (Qres score = 0.85) as shown in Figure 2A. The interactions for FtsQB were extracted from the model (Figure 2B) and compared with the crystal structure (PDB ID: 6H9N) to validate the use of AlphaFold multimer. In the model, interactions between 194 and 256 residues in the periplasmic domain of FtsQ are observed with 52-87 residues of FtsB. Towards the C-terminus, FtsB forms a β-strand that binds to the last β-strand (β-12) of FtsQ to form a continuous β-sheet by antiparallel stacking. FtsB has a loop between the α-helix and β-strand in the C-terminus, interacting with the Tyr248 of FtsQ. A central hydrophobic patch in FtsQ is formed by Y248 and A253, where FtsB latches onto the FtsQ structure. There are aromatic interactions between FtsQ (Y248) and FtsB (residue Phe84), while FtsB Tyr85 is in close contact with the hydrophobic core of FtsQ formed by residues (L226, L230, V254, and W256). All these interactions involving residues 64-87 of FtsB could be validated from the crystal structure [6H9N] of the complex that containing these residues, though there was minor variation in the distances between the side-chains. The additional interactions seen in the model involve the loop between two sheets of FtsQ (residues 194-197) and one face of the helical region of FtsB (residues 48, 52, 56 and 59). These may be due to the orientation of the proteins and not particularly responsible for binding, as deuterium uptake differences cannot confirm these interactions (Kong et al., 2022).

Interestingly, in the multimer model, FtsL also shows parallel β-sheet stacking when bound to the FtsQ periplasmic domain in the only region where all three proteins are conjoined. The periplasmic domain has only two to three hydrogen bonds between FtsQ and FtsL. These interactions could result from FtsB binding to FtsQ with antiparallel β-sheet packing. Although this extension of the β-sheet stacking seems an elegant utilisation of the extended C-terminus region of FtsL which is otherwise disordered, deuterium uptake studies do not provide sufficient validation for this aspect of the model (Kong et al., 2022).

Cell division proteins FtsB and FtsL form a subcomplex prior to their binding to the FtsQ and other cytoplasmic components of the divisome. The helical transmembrane and putative periplasmic domain portion of the FtsBL subcomplex form an intricate web of hydrophobic contacts and hydrogen bonding that maintain the subcomplex (Condon et al., 2018). Through antiparallel β-sheet packing, the FtsBL subcomplex interacts with FtsQ to produce a 1:1:1 heterocomplex, which can dimerize to form a 2:2:2 complex (Villanelo et al., 2011) without any change to the FtsQBL interface in our model. Leucine residues are found in the proximal periplasmic region of FtsL and the distal periplasmic region of FtsB. Due to the possibility of substituting alternative hydrophobic residues, such as isoleucine or valine, for the leucines that make up the zipper motif, this coiled-coil motif may grow along the helix. This complex’s distal and proximal parts, which lack leucine residues, are stabilized by glutamines, valines, and alanines. The last C-terminus residues of FtsB are free because the FtsL periplasmic domain is shorter.

It is believed that FtsW is localized to the septum site by the cytoplasmic region of FtsL; in addition, the predicted multimeric complex also shows interactions with the helical transmembrane and cytoplasmic region of the FtsL (Figure 2C). These proteins interact through two salt bridges in the cytoplasmic domain. Few hydrogen bond interactions occur in the cytoplasm and transmembrane area, while hydrophobic contacts predominate in the transmembrane region. Hence these differential interactions with FtsQ and FtsW can be used to unambiguously assign FtsB and FtsL.

### 3.4 Comparison of FtsQBL interactions in NTD bacteria show similarity to *E. coli*

Multimer model prediction was carried out for FtsQ, FtsL, FtsB, and FtsW in *V. cholerae, M. ulcerans, M. leprae,* and *C. trachomatis* (Figure 2D)*.* In these organisms, only the periplasmic domain of FtsQ was used, as the disordered cytoplasmic regions of the protein interfered with the FtsBLW interactions*.* Predicted remote homologs for FtsB and FtsL in *Mycobacterium sps.* also have long disordered N and C termini regions which were excluded from multimer modeling. Based on the monomer structure superimposition for FtsB and FtsL, residue numbers 78-215 of candidate proteins A0PW54 and Q9CD41; and residue numbers 45-180 of A0PTJ5 and Q7AQC6 were considered for multimer modeling. The quality measure for the accuracy of predicted multimer models is the DockQ score. It measures the quality of the interface and gives a score between 0 and 1. A score ≤ 0.23 is unacceptable for the model and a score=>0.8 is considered a highly accurate model (Basu and Wallner, 2016). The DockQ score for multimer complexes of *V. cholera, M. ulcerans, M. leprae* and *C. trachomatis* are 0.69, 0.40, 0.42, and 0.65 respectively. A comparison was done between *E. coli* multimer model and predicted multimer models from all four organisms to delineate interactions among the proteins as mentioned above.

#### 3.4.1 Comparison of intermolecular interactions of FtsQ, FtsB, and FtsL

As seen in the *E. coli* multimer structure, the β-sheet at its C-terminus domain is the point of interaction between the FtsQ molecule and the FtsB/FtsL heterodimer. The C-terminus residues of FtsB (76–88) and the final beta-strand of FtsQ (251-258) are arranged into a twisted β-sheet and are stabilized by multiple hydrogen bonds. The multimer model of *V. cholerae* shares high similarities and is almost identical to *E. coli*, with many interactions between FtsQ and FtsB, including β-strand and loop formation, conserved in *V. cholerae*.

For *Mycobacterium* sps. and *Chlamydia*, remote homologs of FtsB and FtsL were not distinguished because of their similar structures. These conserved FtsQB secondary structures stacked into antiparallel β-sheet packing in muFtsQ and mlFtsQ were observed with only one of the two candidate proteins that were predicted by the HMM: A0PW54 (muFtsB) and Q9CD41 (mlFtsB) in the periplasmic region of the multimer complex. Secondary structures for the remaining remote homologs muFtsL (A0PTJ5) and mlFtsL (Q9AQC6) were very similar to FtsL protein. They formed parallel β-sheet interactions with muFtsB and mlFtsB. These proteins muFtsL and mlFtsL also show interactions in the periplasmic domain of FtsQ, but the number of interactions are very few compared to muFtsB and mlFtsB. As seen in multimer structures (Figure 2D), remote homologs muFtsB and mlFtsB in *Mycobacterium* sps. do not interact with FtsW in the cytoplasmic region. These interactions are helpful in distinguishing between the FtsL and FtsB remote homologs in *Mycobacterium* sps. In the multimer complex of *C. trachomatis,* ctFtsB (O84041) forms the same β-strand structure in the C-terminus with an extended helix very similar to the ecFtsB homolog. It also forms antiparallel β-sheet packing with the ctFtsQ and does not interact with FtsW in the cytoplasmic domain. The other predicted remote homolog, ctFtsL (O84273), forms a parallel β-sheet packing with ctFtsB.

**FIGURE 3:**
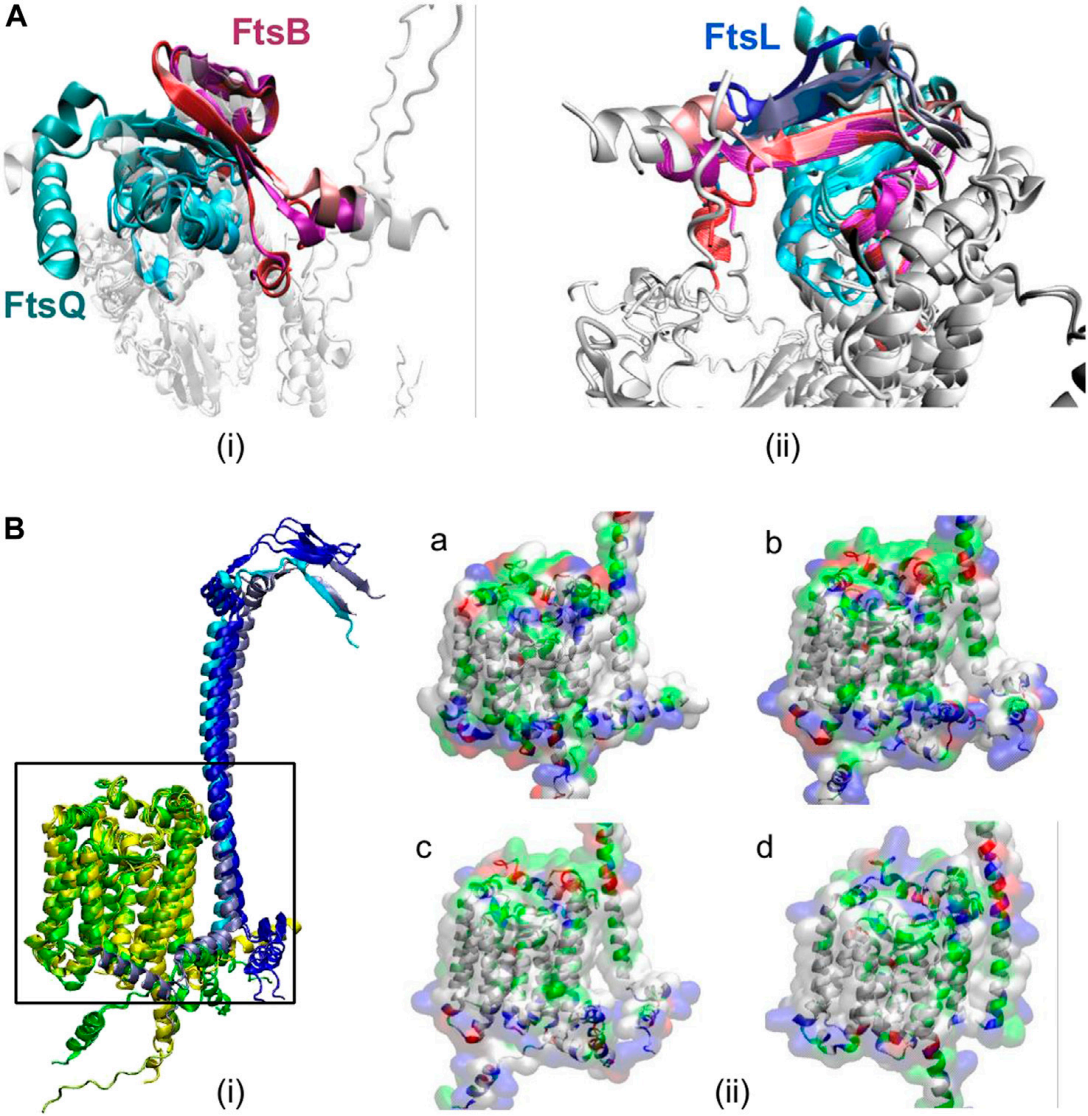
**(A)** Superimposed secondary structures of (i) FtsQB showing antiparallel-beta sheets interaction in the periplasmic region in all organisms. FtsQ is shown in cyan color, and different color variations correspond to all five organisms. FtsB from *E. coli* and *vibrio* are very similar and are shown in red color. FtsB from *Chlamydia* is light pink, and the magenta color represents FtsB from *Mycobacterium ulcerans* and *leprae.* (ii) FtsQBL interactions, here FtsL is shown in ice-blue color for *E. coli* and *vibrio*, cyan color represents FtsL from *Chlamydia* and blue for *Mycobacterium* sps. These anti-parallel beta-sheet interactions between FtsQ, FtsB, and FtsL are highly conserved across all the mentioned organisms. From this superimposition of secondary structures in the periplasmic region, it is easy to distinguish between FtsB and FtsL, as we can see from the structure, but also the number of interactions between FtsB and FtsQ is significantly greater than the interactions with FtsQ/FtsL. **(B)** (i) Superimposed 3-D structures of secondary structures of FtsL (blue) and FtsW (yellow) from all organisms mentioned. FtsL is shown in ice-blue color for *E. coli* and *vibrio*, cyan color represents FtsL from *Chlamydia* and blue for *Mycobacterium sps*. And FtsW is shown in yellow (*E. coli* and *vibrio*), green (*Chlamydia*) and yellow3 (*Mycobacterium* sps). (ii) Surface view of FtsL and FtsW in (a) *V. cholerae,* (b) *M. ulcerans*, (c) *M. leprae*, and (d) *C. trachomatis* colored based on residue type.

Further, the multimer models were superimposed (Figure 3A) with the *E. coli* multimer model based on FtsQ to identify the position of these interacting residues due to their size differences. These superimposed models show proteins muFtsB, mlFtsB, and ctFtsB superimpose with FtsB of *E. coli*. Similarly, muFtsL, mlFtsL, and ctFtsL were superimposed with FtsL of *E. coli*. These multimer models show conserved secondary structure conformations for FtsQ, FtsB, and FtsL remote homologs in *Vibrio cholerae*, *Mycobacterium* sps., and *Chlamydia,* irrespective of their low sequence similarity.

#### 3.4.2 Comparison of intermolecular interactions of FtsL and FtsW

As seen in the *E. coli* multimer complex, the cytoplasmic domain of FtsL is crucial for interaction with FtsW and is not needed for interactions with FtsQ and FtsB. From multimer models, it was observed that the remote homologs muFtsL (A0PTJ5), mlFtsL (Q9AQC6), and ctFtsL (O84273) have an extended cytoplasmic tail that binds to FtsW. And muFtsB, mlFtsB, and ctFtsB are slightly away from FtsW protein and do not interact with FtsW.

Structures of the predicted FtsL homolog (muFtsL, mlFtsL, and ctFtsL) and FtsW subcomplex from the complete multimer model were superimposed (Figure 3B) with the *E. coli* FtsLW complex. The functional region between FtsL and FtsW is highly conserved in *V. cholerae* with respect to *E. coli*, as seen in Figure 3B. Similarly, *M*. *ulcerans* and *M. leprae* also have structural conservation and were superimposed on ecFtsL, but there is an angular shift in the cytoplasmic domain (Figure 3B) which could be a result of long disordered N-terminus. Predicted remote homolog ctFtsL does not have a long extended cytoplasmic domain, and it interlocks with FtsW very tightly.

#### 3.4.3 The number of interactions between FtsQBLW complex proteins are consistent across all organisms

The overall number of interactions between FtsQB, FtsQL, FtsBL, and FtsLW were compared across all the mentioned organisms (Table 2). Multimeric interactions between these proteins were calculated with the help of PDBSum (Laskowski et al., 2018). In *Mycobacterium* sps. and *Chlamydia*, only the periplasmic domain of FtsQ was used for the modeling of FtsQ, FtsB, FtsL, and FtsW as compared to *E. coli* multimer complex where the transmembrane region of FtsK’ and full structure of FtsQ was part of the model. Also, in *Mycobacterium* candidate FtsB (A0PW54, Q9CD41) and FtsL (A0PTJ5, Q7AQC6), only the superimposed functional region with *E. coli* homologs was considered in multimer model building.

**TABLE 2:** This table shows interactions between FtsQ, FtsB, FtsL, and FtsW in all the organisms mentioned in this paper.

	FtsQ:FtsB				FtsQ:FtsL				FtsB:FtsL				FtsL:FtsW			
Organism	IR	SB	HB	NB	IR	SB	HB	NB	IR	SB	HB	NB	IR	SB	HB	NB
*E. coli*	23:19	5	14	175	9:8	0	5	43	49:48	5	19	285	25:27	2	10	146
*V. cholerae (Full Str)*	18:19	7	12	169	5:4	0	0	21	47:46	7	16	271	22:26	1	3	107
*V. cholerae*	19:19	8	14	159	2:1	-	-	7	44:45	7	19	238	21:23	1	4	92
*M. ulcerans*	16:16	3	12	81	4:4	-	-	10	42:42	3	16	193	6:7	-	1	23
*M. leprae*	15:13	3	10	63	3:4	-	-	14	43:42	2	14	220	9:10	-	1	36
*C. trachomatis*	14:13	3	9	90	1:1	-	-	1	44:43	3	17	223	17:15	1	1	78

IR, Interface Residues; SB, Salt Bridge; HB, Hydrogen bond; NB, Non-Bonded Interactions.

Considering major FtsQ and FtsB interactions occur in the periplasmic domain, the number of interactions between FtsQB are almost identical in *E. coli*, and *V. cholera* but are slightly less in *Mycobacterium* sps. and *Chlamydia* (Table 2). The absence of the transmembrane domain of FtsQ in *Mycobacterium* sps. and *Chlamydia* in the model slightly moves the periplasmic domain away from FtsBL while maintaining anti-parallel packing between FtsQB. This was not observed in *V. cholerae* and could result in fewer interactions in *Mycobacterium* sps. and *Chlamydia.*

In *E. coli*, a total of nine and eight interface residues were reported for FtsQ and FtsL. Only 2:2 interfacing residues are present in the periplasmic domain. The number of periplasmic FtsQL interactions in all the mentioned organisms can be considered very similar.

For FtsBL, the number of interacting residues is very close (Table 2). *Mycobacterium sps.* has an extended disordered region on both N and C termini which was excluded from the multimer model prediction.

The interactions in *Mycobacterium* sps. FtsL and FtsW are very few compared to other organisms due to angular shifts in the cytoplasmic region of FtsL (Table 2)*.* This angular shift could be because of the long cytoplasmic and periplasmic disordered regions in FtsL, which reduced the interactions between FtsL and FtsW. This is an additional result to distinguish between FtsB and FtsL in *M. ulcerans*, *M. leprae*, and *C. trachomatis*.

### 3.5 Phylogenetic analysis of FtsQ, FtsB, and FtsL homologs

The structures of all orthologs investigated are readily superimposable, with structurally conserved features which can be associated both to their common fold and to their specificity in binding. However, their sequences are not as conserved. Despite this sequence diversity, proteins with a relatively unique fold like FtsQ can be mapped using hidden Markov models. In the case of FtsB and FtsL, their sequence signatures are not sufficiently specific. In order to explore this further, a phylogenetic analysis of the proteins in context with other known orthologs was performed.

**FIGURE 4:**
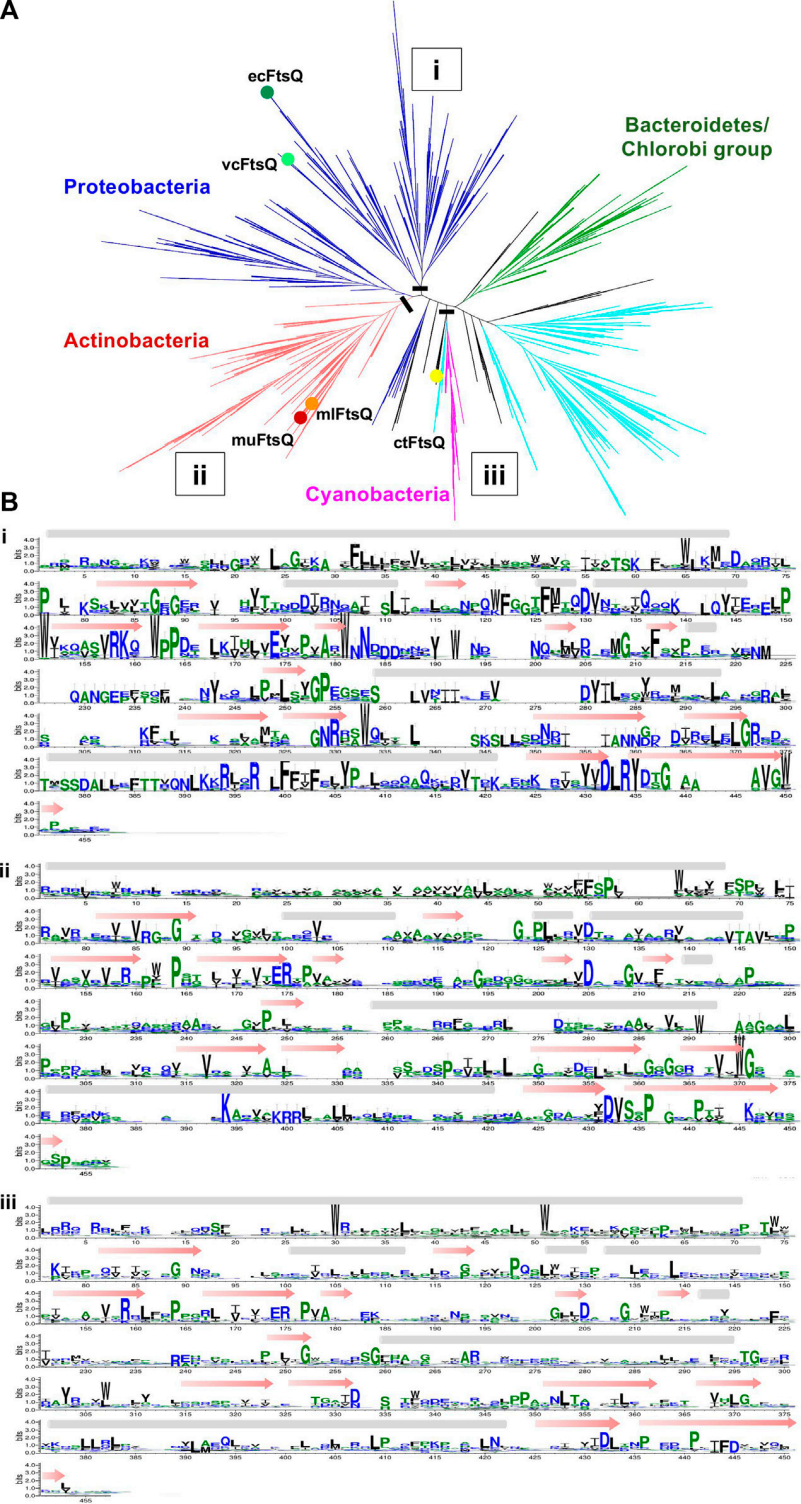
**(A)** Unrooted cladogram represents the phylogenetic clustering of FtsQ from diverse bacterial taxa. The dominant phylum proteobacteria, Firmicutes, actinobacteria, Bacteroidetes/chlorobi group, and cyanobacteria were colored blue, cyan, red, green, and pink respectively. The phyla with less than (0.5%) representation are all colored black. Nodes for the vcFtsQ, muFtsQ, mlFtsQ, and ctFtsQ are highlighted in their respective phyla. Each node shows a divergence of 10% in the phylogenetic tree. **(B)** Sequence logos representative of the multiple alignments built from different phylums/clades in which the FtsQ homologs are present in figure. A black bar shows the location at which tree is cut.

All the predicted remote homologs of FtsQ were mapped to COG1589. Multiple sequence alignments built for the FtsQ ortholog cluster have 4226 sequences from 4163 species. The phylogenetic tree in Figure 4 represents the phylogenetic analysis of FtsQ from diverse bacterial taxa. The most abundant phylum in the COG is proteobacteria consisting of almost 38.7% of the full tree, followed by Firmicutes (24.7%), Actinobacteria (17.2%), and Bacteroidetes/Chlorobi (10%). Nodes for the ecFtsQ, vcFtsQ, muFtsQ, mlFtsQ, and ctFtsQ are found to be clustered within their respective phyla. The clustering of ecFtsQ and vcFtsQ in the same clade is indicative of their functional similarity and evolutionary relationship. Similarly, muFtsQ and mlFtsQ are very closely clustered in the clade representing the Actinobacteria phylum. The remote homolog ctFtsQ was detected in the chlamydiae/verrucomicrobia group, which consists of only 0.5% of the phylogenetic tree. The phylum Firmicutes has a separate cluster, but a few sequences from Firmicutes are shown in Figure 4 to be clustering close to the chlamydiae/verrucomicrobia group (0.5%). A sequence logo representative of the multiple alignments (Figure 4) shows that while the proteobacteria clade has sequence patterns that are clearly visible, the clades with mycobacteria FtsQ and clFtsQ have only aromatic and charged residues that stand out. However, the alignment shows few gaps, and the general pattern of hydrophobicity and hydrophilicity is maintained, which can explain the easy identification of orthologs across the bacterial kingdom using hidden Markov models.

The HMM fails to distinguish FtsB and FtsL unambiguously. In order to investigate why FtsB is occasionally scored higher than the actual ortholog with an HMM prepared from ecFtsL, the clustering patterns for the sequences of both orthologs were observed together. The FtsB COG2919 has 4644 sequences from 3857 species, out of which 76 are labeled as FtsL. There are two COGs for the FtsL protein: COG3116, which contains 999 sequences from 997 proteobacteria species, and COG4839 which contains 549 sequences from an identical number of species, 98.2% from the Firmicutes phylum. Two proteins ecFtsL and vcFtsL were mapped to COG3116. But muFtsL and mlFtsL did not map to either of them. Surprisingly, muFtsL was found in COG related to penicillin-binding proteins but could be a false positive incorrectly clustered due to the large disordered regions present in these sequences. The other protein mlFtsL maps to FtsB COG, which is understandable as this COG contains many FtsL proteins. As in the case of ctFtsB, ctFtsL is also missing in the database.

**FIGURE 5:**
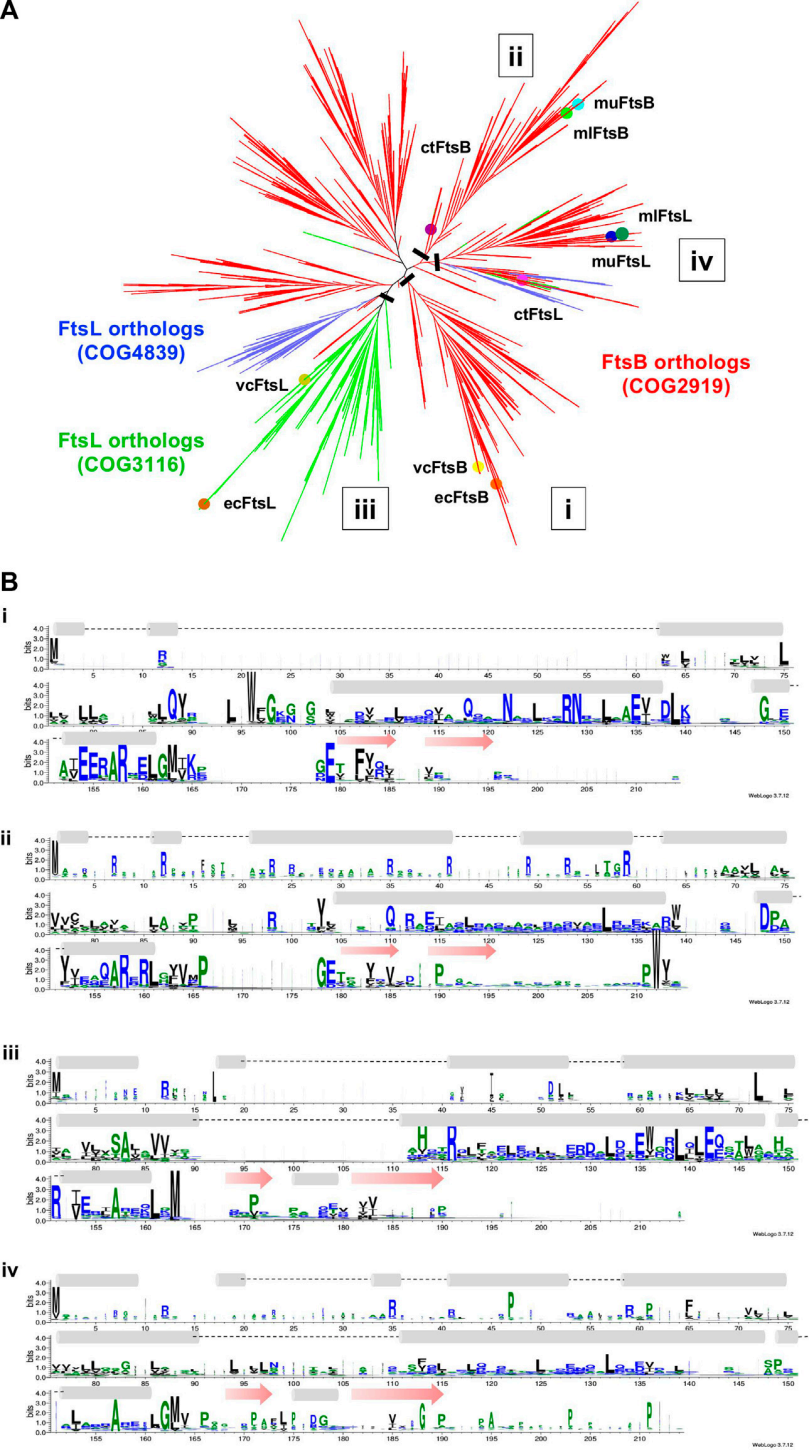
**(A)** Unrooted cladogram represents the phylogenetic analysis of FtsB and FtsL from diverse bacterial taxa. In the phylogenetic tree red subtree represents the FtsB COG2919, green and blue represent the FtsL COG3116 and COG4839. Nodes for FtsB and FtsL homologs are highlighted for all five organisms. **(B)** Sequence logo for FtsB and FtsL homologs built separately for the two clades in which they are present. A black bar shows the location at which tree is cut.

All the sequences for FtsB and FtsL from three COGs were taken together, and in addition to this, individual sequences for muFtsL, ctFtsL, and ctFtsB were also added to this dataset. As shoen in Figure 5 ecFtsB and vcFtsB are present in the FtsB orthologs cluster, and ecFtsL and vcFtsL are in the same clade in the FtsL orthologs cluster. Homologs for FtsB and FtsL for *Mycobacterium* were found in a different subtree that bifurcates into two clades with muFtsB and mlFtsB present in one clade, muFtsL and mlFtsL present on the other. The ctFtsB is present in the same clade as *Mycobacterium* FtsB but is distant from it. Homolog ctFtsL is present in the clade, which consists of a few taxa from all three COGs. The sequence alignment (Figure 5) for these clades is instructive: only the portion of the common pattern in the amphipathic helix is stacked together. Both proteins have additional domains of unknown function fused to each of the N and C termini of the core regions that interfere with sequence patterns responsible for functional specificity–for FtsL, the N-terminus region that interacts with FtsW and for FtsB, the C-terminus region that interacts with FtsQ.

## 4 Discussion

In this paper, homology-based methods were used in a sequential manner, to identify the homologs of the FtsQBL complex from *E. coli* in four different NTD species that represent both the genome diversity within bacteria phyla and incompletely characterized organisms of potential importance. The pathogen *V. cholerae* has divisome components very similar to *E. coli* and all three homologs were easily identified using sequence-sequence similarity with BLAST. However, as the sequence diversity increases, sequence-sequence comparison methods lose their sensitivity. Increased sensitivity is provided with the profile-sequence comparison HMMER, using profiles created from known orthologs of the query protein, and Profile-Profile (HMM) comparison methods with HH-suite, using profiles built from a preprocessing step of extracting similar sequences from uniprot20, a version of the Uniprot database with sequence redundancy at 20%. HHsearch method was able to find remote homologs of the FtsQBL complex in *Mycobacterium sps* and *Chlamydia.* The potential remote homologs for FtsQ identified in this study for *M. ulcerans, M. leprae*, and *C. trachomatis* are A0PTI5, Q9CCE5, and O84769*,* respectively. We identified the same protein as ctFtsQ that Ouellette et al., 2015 (Ouellette et al., 2015) reported in their study. Remote homologs of FtsB and FtsL in *Mycobacterium sps.* and *Chlamydia* were not distinguishable with this method, therefore structural modeling of proteins was done using AlphaFold to resolve the ambiguity. These homologs were still ambiguous due to their similar domain and structural fold. To further characterize their function, their multimeric interactions with FtsQ and FtsW were used to distinguish the orthologs. The combined use of fold and protein-protein interactions could be used to map A0PW54 (muFtsB), Q9CD41 (mlFtsB), and O84041 (ctFtsB) as remote homologs of ecFtsB because of their interactions with the periplasmic domain of FtsQ and A0PTJ5 (muFtsL), Q9AQC6 (mlFtsL), and O84273 (ctFtsL) as remote homologs of ecFtsL due to their cytoplasmic domain interaction with FtsW. BLAST alignment for predicted homologs of FtsB and FtsL in *Mycobacterium sps.* with the *M. smegmatis* homologs) (Wu et al., 2018) provide further validation of our approach. The FtsB in *M. smegmatis* is a fusion protein with a domain of unknown function (DUF501) attached to its C-terminus - which is an immediate neighbor of predicted FtsB homologs in both the *Mycobacterium* species. The DUF501 domain maps to residues 227-388 of msFtsB while the muFtsB and mlFtsB map to 1-197 and 1-214 residues of msFtsB. These patterns made it difficult to differentiate between FtsB and FtsL on the basis of phylogeny. Ouellette et al. (2015) (Ouellette et al., 2015) reported *ct*FtsL homolog with gene name CT_271 (UniProt ID: O84273), which is the same protein that we characterized as ctFtsL because of its interactions with FtsW in the *C. trachomatis* multimer protein complex. Multimeric interactions played a very important role in successfully characterizing the FtsB and FtsL in M*ycobacterium sps. and Chlamydia*. The proteins identified as ctFtsQ and ctFtsL are identical to those previously reported from the experimental findings validates our hypothesis and methodology. In addition, we found a potential remote homolog for ctFtsB.

The hidden Markov model is a mathematical representation of the multiple alignment of sequences in a gene family. Its efficacy is dependent on the quality of clustering sequences into both phylogenetic relationships and gene families which can be used to generate sequence signatures. Common choices for annotation would be the Panther database - which has gene families curated and clustered from 143 genomes into gene families (Thomas et al., 2022) and Inparanoid (Sonnhammer and Östlund, 2015), which identifies and clusters orthologs from pairwise species comparisons but is more focused on Eukaryotic genomes. The EGGNOG database, created by non-supervised clustering of sequences from all-versus-all pair-wise local alignments, allows for choice in selecting sequences for a gene family at the level of the complete COG, or segmented use at a finer taxonomic level providing some user control on specificity. Profile-profile methods are commonly used to detect diversified proteins with a common fold, and hence ortholog specificity can be even lower with the use of these methods. The HH-suite programs are distributed with the UniRef30 database, created by clustering sequences from the UniRef database with 30% similarity. The use of this default database generated known false positive hits for FtsB and FtsL, especially from the *Mycobacteria* species, which have a number of proteins containing domains of unknown function. Both these proteins functionally interact by forming a coiled-coil, a common motif in many protein-protein interactions. The results described in this paper use an earlier version of a clustered database, uniprot20, that was more specific.

Exploiting the FtsQBL complex proteins interactions with one another and other proteins may aid in discovering drugs that inhibit bacterial growth because of their role in the divisome assembly. In this paper, we modeled the FtsQBLW multimer complex in *M. ulcerans, M. leprae,* and *C. trachomatis* and identified their key interactions to shed light on the mechanism of their binding as well as to identify the areas that should be the focus of inhibitors. The interactions between FtsQ and FtsB play an important role in the formation of the FtsQBL complex because FtsB and FtsL bind to each other and then bind to FtsQ with the help of the FtsQB periplasmic domain, which makes the interactions between FtsQB an excellent target for cell division inhibitors. A previous study (Kureisaite-Ciziene et al., 2018) provides experimental findings about the critical role of Tyr248 in the formation of EcFtsQBL complex that shows that the mutations (Y248W and Y248K) have a dominant-negative effect on the FtsQB binding and function. This residue position is on the loop connecting the last two β-sheets towards the C-terminus. The Tyr248 is highly conserved in proteobacteria but has been replaced with Serine in *Mycobacterium* sp. and with Cysteine in *Chlamydia*. These residues can be exploited to act as a specific drug target for inhibition of the FtsQB subcomplex in respective pathogens.

In this paper, our aim is to predict homologs from model organisms like *E. coli* for evolutionary distant species with high confidence. Sequence diversity prevents the identification of the remote homologs in distant species with traditional homology-based methods like BLAST. The more sensitive methods like Profile-Profile comparison along with structural modeling of proteins using AlphaFold - especially in a multimer complex - can be used to assign a specific function to remote homologs that otherwise cannot be easily annotated from traditional sequence analysis methods. The remote homologs we identified are identical to *in vivo* studies which show that the methodology used is capable of detecting homologs in distantly related species, while providing the scope to directly apply structure comparison techniques to study the ortholog. The application of deep learning has recently been made to directly annotate function from a protein’s sequence (Bileschi et al., 2022), and since been applied to the uniprotKB database with higher accuracy, functionally identifying ctFtsQ and ctFtsL, but is still unable to annotate the FtsB and FtsL homologs from *Mycobacterium* and ctFtsB. This technique provides an faster and more accurate alternative to traditional methods in mapping function to orthologs, but is silent on both sequence and structural features responsible for the proteins function which can be derived from conserved sequence signatures and the proteins structure.

## Data Availability

The datasets presented in this study can be found in online repositories. The names of the repository/repositories and accession number(s) can be found in the article/Supplementary Material.

## Nomenclature

### Resource identification initiative

NCBI Datasets: (RRID: SCR_022569)
NCBI BLAST: (RRID: SCR_004870)
HMMER: (RRID: SCR_005305)
Uniprot: (RRID: SCR_002380)
eggNOG: (RRID: SCR_002456)
VMD: (RRID: SCR_001820)
PDBsum: (RRID: SCR_006511)
